# Genetic Diversity and Haplotype Architecture of Non‐Classical HLA Class I Genes in the Japanese Population

**DOI:** 10.1111/tan.70898

**Published:** 2026-08-10

**Authors:** Ikue Ito‐Naito, Seik‐Soon Khor, Tetsuya Sato, Shunichi Wakabayashi, Daisuke Kinoshita, Naoya Kubo, Yosuke Omae, Katsushi Tokunaga, Hiroshi Hayashi

**Affiliations:** ^1^ H.U. Group Research Institute G.K. Tokyo Japan; ^2^ Genome Medical Science Project National Center for Global Health and Medicine, Japan Institute for Health Security Tokyo Japan; ^3^ Singapore Centre for Environmental Life Sciences Engineering, Nanyang Technological University Singapore Singapore

**Keywords:** four‐field resolution, haplotype, HLA‐E, HLA‐F, HLA‐G, Japanese, long‐read sequencing

## Abstract

The non‐classical Class I genes (HLA‐E, HLA‐F and HLA‐G) show limited protein‐level diversity; however, full‐length analyses that include regulatory and noncoding regions have not been sufficiently performed in East Asian populations. We designed new primers for long‐range PCR and conducted PacBio SMRT sequencing to generate full‐length gene sequences of HLA‐E, HLA‐F and HLA‐G, including 5′ and 3′ untranslated regions (UTRs) as well as coding and intronic regions in 531 healthy Japanese individuals. These data enabled the full phasing of HLA‐E, HLA‐F and HLA‐G, including upstream and downstream variants, at four‐field resolution. We identified 24 HLA‐E alleles, 30 HLA‐F alleles and 19 HLA‐G alleles, including a total of 36 novel (10 HLA‐E, 19 HLA‐F and 7 HLA‐G) and 16 extended alleles (13 HLA‐E, 1 HLA‐F and 2 HLA‐G). Protein‐level diversity for HLA‐E was limited, with E*01:03 and E*01:01 predominating. For HLA‐F, despite the predominance of F*01:01 (96.7%), noncoding diversity at four‐field resolution was abundant; the expression‐linked rs2523405‐T allele phased with F*01:01:02 and F*01:01:06, as well as with a subset of F*01:01:01 sub alleles. For HLA‐G, consistent with previous reports, four‐field resolution alleles and UTR variants formed fully co‐segregating haplotypes. Linkage disequilibrium (LD) analysis at four‐field resolution showed no LD between HLA‐E and either HLA‐F or HLA‐G, whereas moderate LD was observed between HLA‐F and HLA‐G; the strongest signal was driven by F*01:01:02:09, which forms a haplotype with G*01:01:03:03. In summary, full‐gene long‐read sequencing clarified allele frequencies of non‐classical Class I genes in the Japanese population, delineated phasing in regulatory regions and refined the LD structure between HLA‐F and HLA‐G by resolving four‐field haplotypes. These data constitute an important reference for immunogenetic research, transplantation and disease‐association studies in the Japanese population.

## 1 Introduction

The non‐classical HLA Class I (HLA‐Ib) genes, HLA‐E, ‐F and ‐G, exhibit gene and protein structures similar to those of the classical HLA Class I (HLA‐Ia) genes, HLA‐A, ‐B and ‐C, while displaying limited genetic diversity.

HLA‐E is expressed at low levels on most human cells and regulates natural killer (NK) cells and T cells through signalling mediated by the inhibitory heterodimeric receptor CD94/NKG2A and its activating counterpart CD94/NKG2C [1, 2, 3]. Signal peptides derived from HLA Class I allotypes are presented as nonamer peptides bearing valine at position 1 and leucine at position 9 (VL9), which are critical for HLA‐E binding and CD94/NKG2 receptor engagement. HLA‐E is represented by two major allele groups, E*01:01 and E*01:03, across diverse populations worldwide [4, 5, 6, 7]. The difference between these alleles is located at amino acid position 107, with arginine in E*01:01 and glycine in E*01:03 and compared with E*01:01, E*01:03 exhibits higher peptide‐binding affinity and greater protein thermal stability [8], resulting in increased cell‐surface expression [9]. Previous studies have shown that E*01:03 is the most frequent *HLA‐E* allele in East Asian populations [5, 6, 7]. Within the E*01:03 group, two synonymous alleles, E*01:03:01 and E*01:03:02, are commonly reported; they differ by a single nucleotide substitution in exon 2 (g.424 C > T). Notably, in Asian populations, E*01:03:01 occurs at a higher frequency than E*01:03:02, in contrast to patterns observed in other populations [5]. E*01:12:01:01 has been reported from Asian samples; however, its allele frequency within Asian populations has not yet been documented [10].

HLA‐F is expressed at two structural forms: folded heavy chains complexed with β2‐microglobulin (β2m) and peptide, and open conformers (without β2m and peptide) [11]. These structural differences influence receptor engagement, as HLA‐F can bind KIR3DS1, KIR3DL2 and KIR2DS4 on NK cells [11], with the open conformer exhibiting markedly higher affinity for KIR3DS1 [12]. Although HLA‐F is expressed in immune cells such as B cells and monocytes [13], as well as in lung epithelial cells and platelets [14], it remains predominantly intracellular, and strong cellular activation is required for its cell‐surface expression or secretion [14, 15]. Compared with HLA‐E and HLA‐G, whose functional roles are increasingly supported by both mechanistic and clinical studies, the role of HLA‐F remains less well characterised, with limited supporting evidence. In the East Asian population, HLA‐F is predominantly represented by F*01:01, with a frequency of 97.7%, substantially higher than that reported in other populations [7]. High‐resolution allele frequency data for HLA‐F have been reported for the Brazilian [16], the Beninese Toffin [17], a German cohort [18], as well as Asian, African American and Caucasian samples [19]. Previous studies in the Brazilian and the German reported that F*01:01:01:01, F*01:01:01:08 and F*01:01:01:09 were relatively frequent within the F*01:01 group, followed by F*01:03:01. However, because the Asian sample size was limited, no study has yet provided sufficient data to discuss four‐field allele frequencies in East Asian populations.

HLA‐G plays an important role in pregnancy, being expressed in extravillous trophoblasts of the placenta and playing a key role in establishing maternal–foetal immune tolerance. In addition, HLA‐G contributes to immune regulation and functions as an immune checkpoint and is expressed in several immune cell types, including monocytes and regulatory T cells [20, 21]. Seven HLA‐G isoforms have been identified, generated through alternative mRNA splicing; four are membrane‐bound, whereas the remaining isoforms are soluble [22]. HLA‐G exerts its immunomodulatory effects through interactions with its receptors, such as LILRB1 and LILRB2 on lymphoid and myeloid cells [23], and KIR2DL4 on NK cells [24]. In the Japanese population, the *HLA‐G* locus is largely represented by the two alleles G*01:01 and G*01:04 [25, 26], and the most common four‐field alleles within these groups are G*01:01:01:01 and G*01:04:01:01 [27, 28]. The 14‐bp insertion polymorphism rs371194629 and rs1063320 in exon 8 of the HLA‐G 3′ untranslated region (3′ UTR) have been suggested to have functional significance. These variants are located outside the regions registered in the IPD‐IMGT/HLA Database [29]. In this study, our primer set was designed to include the exon 8 region encompassing the 14‐bp insertion, enabling long‐read sequencing–based phasing that resolves these 3′ UTR variants onto specific HLA‐G alleles.

Within the HLA Class I region of chromosome 6 (6p21.3), the HLA‐E locus is positioned between HLA‐A and HLA‐C, whereas HLA‐F and HLA‐G are located in proximity to HLA‐A. Reflecting these genomic distances, HLA‐E shows little link with HLA‐F and HLA‐G, while a moderate linkage disequilibrium (LD) is observed between HLA‐F and HLA‐G [19].

The HLA‐Ib genes, which exhibit low variability and relatively large distances between polymorphic sites compared with HLA‐Ia genes, present challenges for short‐read sequencing approaches due to phase ambiguity. Furthermore, LD patterns among HLA‐E, HLA‐F and HLA‐G at full‐gene resolution have not yet been comprehensively characterised.

In this study, we aimed to elucidate high‐resolution, four‐field allele frequencies of HLA‐Ib genes in the Japanese population by applying a highly accurate and optimised analysis pipeline with a long‐read sequencing platform (PacBio Sequel and Sequel IIe systems), to clarify the relationships between four‐field alleles and variants located upstream or downstream of regions registered in the IPD‐IMGT/HLA Database, and to define haplotype structures of HLA‐E, HLA‐F and HLA‐G at the four‐field level.

## 2 Materials and Methods

### 2.1 DNA Samples

DNA samples were obtained from the DNA Bank of the Japanese Collection of Research Bioresources (https://cellbank.nibiohn.go.jp/english). These samples were obtained from healthy adult volunteers from various regions of Japan and are considered representative of the Japanese general population [30]. We utilised DNA samples from 367 males and 164 females, ranging in age from 34 to 74 years at the time of collection.

### 2.2 PCR Amplification

**FIGURE 1:**
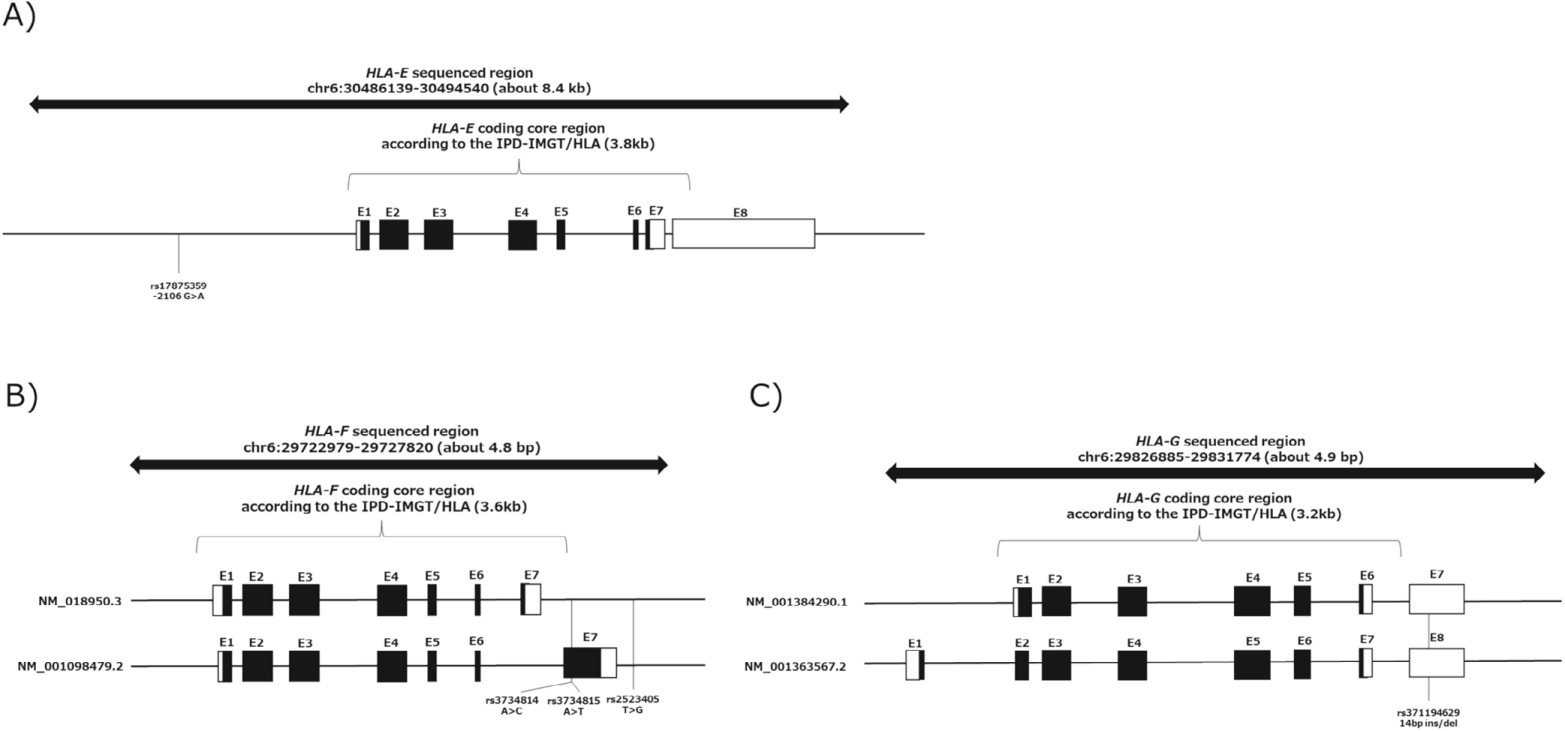
Schematic view of PCR products generated by the designed primers. The amplified regions of the HLA‐E (A), HLA‐F (B) and HLA‐G (C) genes are shown on the hg38 genome coordinates along with their respective target regions.

All primers were modified at the 5′ end with an amino modifier C6 (/5AmMC6/). PacBio universal sequences were added to the 5′ end of each primer. Each of the HLA‐E, HLA‐F and HLA‐G genes was independently amplified as a single long‐range PCR amplicon. The HLA‐E amplicon was approximately 8.4 kb in length (Figure 1A), spanning from the extended promoter region to the complete 3′ UTR. The HLA‐F locus (approximately 4.8 kb; Figure 1B) and the HLA‐G locus (approximately 4.9 kb; Figure 1C) amplicons spanned from the 5′ UTR to 3′ UTR. Detailed primer designs for long‐range PCR and PCR cycling conditions are provided in the Supporting Information.

### 2.3 Library Preparation and Sequencing

After purification of the first PCR products, a second barcoded PCR was performed using PacBio Barcoded Universal Primers (BUP). Libraries for sequencing were constructed from pooled DNA for each sample using the SMRTbell Express Template Prep Kit (Pacific Biosciences of California Inc., Menlo Park, CA, USA) according to the manufacturer's instructions. Sequencing was performed on the PacBio Sequel or Sequel IIe system (Pacific Biosciences of California Inc., Menlo Park, CA, USA). Details of the library preparation are provided in the Supporting Information.

Candidates for novel or extended alleles across HLA‐E, HLA‐F and HLA‐G were validated by performing independent PCR reactions, followed by library preparation and sequencing them on a MiSeq system (Illumina, San Diego, CA, USA) using 251 bp paired‐end reads with the MiSeq Reagent Kit v2, following the manufacturer's instructions. To meet the IPD‐IMGT/HLA Database submission requirements, all candidate alleles were confirmed by sequencing products derived from at least two independent PCR reactions.

### 2.4 Data Analysis

Subreads were generated from raw polymerase reads, and circular consensus sequencing (CCS) reads were produced using SMRT Link package (ccs, version 8.0.1) with the heteroduplex algorithm option (hd‐finder) enabled. Because subreads are derived from single‐strand sequencing, they are not affected by heteroduplex formation; however, they exhibit a relatively high error rate. Therefore, subreads were used to generate highly accurate, phased and full‐length consensus sequences using the Long Amplicon Analysis (LAA, version 2.5.0). In parallel, CCS reads, which provide higher single‐molecule read accuracy than subreads, were processed using PacBio Amplicon Analysis (pbAA, version 1.0.3) to generate highly accurate consensus sequences and corresponding FASTQ files. However, unlike subreads, CCS reads can still be influenced by heteroduplexes; therefore, LAA‐derived consensus sequences were additionally generated and used to support correct phasing and allele assignment. All generated datasets were analysed using NGSengine (GenDx, Utrecht, the Netherlands). HLA‐F contains a short tandem repeat (STR) region composed of TG repeats at position 3097, and differences in the number of TG repeats alone can result in distinct HLA‐F alleles. The repeat structures were genotyped using the Tandem Repeat Genotyping Tool (TRGT, version 0.8.0). The data analysis workflow is summarised in Figure S1.

### 2.5 Linkage Disequilibrium Analysis

Visualisation of haplotype structures among HLA‐E, HLA‐F and HLA‐G alleles was performed using Disentangler (http://kumasakanatsuhiko.jp/projects/disentangler/). HLA‐E, HLA‐F and HLA‐G haplotype frequencies were estimated using PyPop software (version 1.4.1) and BIGDAWG (version 2.1), both implementing maximum likelihood estimation based on the expectation–maximisation (EM) algorithm.

Pairwise LD between HLA‐F and HLA‐G four‐field resolution alleles was assessed using PLINK v1.9 (build 1.90b8). Alleles with a minor allele frequency (MAF) ≥ 4% were retained. LD coefficients (*D′* and *r* ^2^) were estimated using the ‐‐ld command in PLINK.

## 3 Results

Using newly designed long‐range PCR primers that comprehensively amplified full‐length HLA‐E, HLA‐F and HLA‐G, we sequenced 531 Japanese samples with SMRT sequencing technology. Across the HLA‐Ib genes, we identified 24 HLA‐E, 30 HLA‐F and 19 HLA‐G alleles, including a total of 35 novel (10 HLA‐E, 18 HLA‐F and 7 HLA‐G) and 16 extended alleles (13 HLA‐E, 1 HLA‐F and 2 HLA‐G). Four‐field resolution diversity was higher than previously recognised and long‐read sequencing enabled accurate phasing of upstream and downstream variants.

### 3.1 HLA‐E Diversity in Japan

#### 3.1.1 Novel and Extended HLA‐E Alleles

We identified 10 novel and 13 extended full‐length HLA‐E alleles, most of which differed in non‐coding regions (Table 1). Novel alleles were detected at a frequency of 1.1%. A higher frequency (1.8%) has been reported in a previous full‐gene sequencing study by the Anthony Nolan Research Institute [6], which analysed over 6000 samples, including patients undergoing allogeneic HCT and unrelated donor candidates for verification typing, although differences in cohort composition should be noted. A synonymous variant, E*01:03:42 (c.528C > T), was also identified in the coding region.

**TABLE 1:** HLA‐E allelic frequencies.

Allele	Number of observations (2n = 1062)	Frequency (2n = 1062)	GenBank accession number	IPD‐IMGT/HLA Database accession number	Status
E*01:01:01:01/20	125	11.77%	‐/PQ317186	‐/HWS10092511	Extended
E*01:01:01:02	1	0.09%	—	—	—
E*01:01:01:03	155	14.60%	PP493262	HWS10069973	Extended
E*01:01:01:06	5	0.47%	PP493265	HWS10069979	Extended
E*01:01:01:11	3	0.28%	PQ317185	HWS10092513	Extended
E*01:01:01:12	7	0.66%	PP533478	HWS10070079	Extended
E*01:01:01:50	3	0.28%	OP584931	HWS10064171	Novel
E*01:01:01:55	1	0.09%	PP591964	HWS10070225	Novel
E*01:01:01:65	1	0.09%	PQ479190	HWS10093195	Novel
E*01:03:01:01	254	23.92%	—	—	—
E*01:03:01:02	98	9.23%	OR991102	HWS10068771	Extended
E*01:03:01:04	92	8.66%	PP493263	HWS10069975	Extended
E*01:03:01:14	1	0.09%	OP584933	HWS10064175	Novel
E*01:03:01:15	1	0.09%	OP584934	HWS10063372	Novel
E*01:03:02:01/27/32	277	26.08%	‐/PQ317183/PQ317184	‐/HWS10093159/HWS10092509	Extended
E*01:03:02:06	5	0.47%	PP580105	HWS10070171	Extended
E*01:03:02:34	1	0.09%	OP584932	HWS10064173	Novel
E*01:03:02:35	1	0.09%	OP595161	HWS10068599	Novel
E*01:03:02:36	1	0.09%	PP591963	HWS10070223	Novel
E*01:03:02:42	1	0.09%	PQ479191	HWS10093197	Novel
E*01:03:05:01	2	0.19%	PP580106	HWS10070173	Extended
E*01:03:42	1	0.09%	PQ479189	HWS10093193	Novel
E*01:12:01:01	1	0.09%	PP580107	HWS10070173	Extended
E*01:12:01:02	25	2.35%	PP493264	HWS10069977	Extended

*Note:* ‐: Not‐Applicable.

#### 3.1.2 Repeat Region Ambiguities (RRA)

Two thymine homopolymer tracts in intron 4 generated repeat region ambiguities (RRA) in four‐field assignments (e.g., E*01:01:01:01/20 and E*01:03:02:01/27/32) [31]. Given PCR slippage and technical limitations in the exact determination of homopolymer lengths, we reported RRA strings rather than forcing discrete repeat lengths (Table 1).

#### 3.1.3 HLA‐E Allele Distribution

In this cohort, three protein‐level HLA‐E alleles were observed: E*01:03 (69.2%), E*01:01 (28.3%) and E*01:12 (2.5%). E*01:04, previously reported in Japan [32], was not detected in any sample in our Japanese cohort. At the four‐field resolution, E*01:03:02:01/27/32 (26.1%) and E*01:03:01:01 (23.9%) were most frequent, followed by E*01:01:01:03 (14.6%) (Table 1). Within E*01:03:01, E*01:03:01:01 predominated over E*01:03:01:02 and E*01:03:01:04. For E*01:12, E*01:12:01:02 (submitted based on Japan and China derived cell lines [33]) accounted for 2.4%, whereas E*01:12:01:01 (submitted by a group in China [12]) was rare (0.1%). In previous studies, E*01:01:01:01 was reported as the most frequent allele in cohorts of predominantly European ancestry and in the Beninese Toffin [6, 17].

#### 3.1.4 Genotype Distribution

Protein‐level homozygosity was 5.5% (E*01:01) and 34.5% (E*01:03), and homozygosity at four‐field resolution totaled 17.9% (six genotypes; Table S1). The most common heterozygous genotypes were E*01:03:01:01, E*01:03:02:01/27/32 (18.3%) and E*01:01:01:03, E*01:03:02:01/27/32 (8.5%). Several frequent genotype combinations were phase‐ambiguous by short‐read sequencing but were resolved by long‐read phasing, such as E*01:03:01:04, E*01:03:02:01/27/32 versus E*01:03:01:01, E*01:03:02:06.

#### 3.1.5 Co‐Segregation of HLA‐E Alleles With Flanking Variants

Because our *HLA‐E* sequencing strategy also covered upstream regions (Figure 1A), we examined co‐segregation between four‐field HLA‐E alleles (≥ 1% frequency) and previously reported upstream and downstream SNVs [17, 34]. For E*01:01:01:01, rs17875359 (g.–2106G > A) did not exhibit fixed co‐segregation in this cohort, consistent with prior observations that this locus is not tied to a single allele. In this cohort, the G and A alleles occurred at comparable frequencies, whereas previous studies in the Brazilian population reported the G allele to be 8.75‐fold more frequent.

### 3.2 HLA‐F Diversity and Expression‐Linked Structure

#### 3.2.1 Novel and Extended HLA‐F Alleles

Full‐length sequencing identified 19 novel HLA‐F alleles. Several of the newly registered alleles were distinguishable solely by variation in TG repeat length. In total, 20 alleles (novel or extended) have been submitted to GenBank and the IPD‐IMGT/HLA Database (Table 2).

**TABLE 2:** HLA‐F allelic frequencies.

Allele	Number of observations (2n = 1062)	Frequency (2n = 1062)	GenBank accession number	IPD‐IMGT/HLA Database accession number	Status
F*01:01:01:01	195	18.36%	—	—	—
F*01:01:01:08	86	8.10%	—	—	—
F*01:01:01:09	275	25.89%	—	—	—
F*01:01:01:11	3	0.28%	—	—	—
F*01:01:01:13	70	6.59%	OR855420	HWS10068395	Extended
F*01:01:01:17	10	0.94%	—	—	—
F*01:01:01:44	6	0.56%	PP616720	HWS10070281	Novel
F*01:01:01:48	8	0.75%	PQ230459	HWS10092199	Novel
F*01:01:01:49	1	0.09%	PQ230460	HWS10092201	Novel
F*01:01:01:50	1	0.09%	PQ230461	HWS10092203	Novel
F*01:01:01:51	11	1.04%	PQ230462	HWS10092205	Novel
F*01:01:01:52	6	0.56%	PQ230464	HWS10092209	Novel
F*01:01:01:53	1	0.09%	PQ261165	HWS10092297	Novel
F*01:01:01:54	2	0.19%	PQ261166	HWS10092299	Novel
F*01:01:01:55	1	0.09%	PQ329343	HWS10092567	Novel
F*01:01:01:56	1	0.09%	PQ329345	HWS10092577	Novel
F*01:01:01:57	1	0.09%	PQ329347	HWS10092581	Novel
F*01:01:01:58	4	0.38%	PQ329349	HWS10092585	Novel
F*01:01:02:09	51	4.80%	—	—	—
F*01:01:02:10	218	20.53%	—	—	—
F*01:01:02:15	1	0.09%	PQ261167	HWS10092301	Novel
F*01:01:02:16	1	0.09%	PQ329348	HWS10092583	Novel
F*01:01:06	74	6.97%	—	—	—
F*01:03:01:01	4	0.38%	—	—	—
F*01:05:01:02	20	1.88%	PQ230463	HWS10092207	Novel
F*01:14:01:01 N	4	0.38%	—	—	—
F*01:14:01:02 N	1	0.09%	PQ329346	HWS10092579	Novel
F*01:21	4	0.38%	PP616719	HWS10070279	Novel
F*01:22	1	0.09%	PQ329344	HWS10092569	Novel
F*01:24	1	0.09%	PQ479192	HWS10093157	Novel

*Note:* ‐: Not‐Applicable.

Several of the newly registered alleles were distinguishable solely by variation in TG repeat length. These differences represent a well‐defined STR structure and were resolved using TRGT, allowing accurate determination of repeat number without the need for RRA representation. Among the newly registered alleles, F*01:01:01:51 differed from F*01:01:01:08, F*01:01:01:52 from F*01:01:01:09, F*01:01:01:58 from F*01:01:01:01, F*01:01:02:15 from F*01:01:02:09 and F*01:01:02:16 from F*01:01:02:15, with each pair differing only by a single TG repeat. In this study, aside from these allele pairs, the only additional case attributable solely to differences in TG repeat length was the pair F*01:01:01:01 and F*01:01:01:17.

#### 3.2.2 HLA‐F Allele Distribution and Common Genotypes

Seven protein‐level HLA‐F alleles were observed, with F*01:01 predominant (96.7%). At the three‐field resolution, F*01:01:01 (64.2%), F*01:01:02 (25.5%) and F*01:01:06 (7.0%) constituted the major subtypes. At four‐field resolution, the most frequent alleles were F*01:01:01:09 (25.9%), F*01:01:02:10 (20.5%) and F*01:01:01:01 (18.4%) (Table 2).

A previous study of cohorts of predominantly European ancestry and a Brazilian cohort reported that F*01:01:01:01 and F*01:01:01:09 were common in both cohorts. In contrast, F*01:03:01:01 was not among the most frequent alleles in this Japanese cohort.

Common genotypes included the homozygotes F*01:01:01:09, F*01:01:01:09 (6.0%) and F*01:01:02:10, F*01:01:02:10 (4.5%), as well as the heterozygotes F*01:01:01:09, F*01:01:02:10 (6.4%) and F*01:01:01:01, F*01:01:01:09 (6.0%) (Table S2).

#### 3.2.3 Regulatory‐Region Phasing and Expression‐Linked Variants

The rs2523405‐T allele co‐segregated with F*01:01:02 and F*01:01:06. Notably, among the F*01:01:01 subtypes, only F*01:01:01:09 was phased with rs2523405‐T (Table 3).

**TABLE 3:** Phased downstream SNVs of the HLA‐F allele observed at frequencies higher than 1%.

Allele	Frequency (2n = 1062)	rs3734814	rs3734815	rs2523405
F*01:01:01:01	18.36%	A	A	G
F*01:01:01:08	8.10%	A	A	G
F*01:01:01:09	25.89%	A	A	T
F*01:01:01:13	6.59%	A	A	G
F*01:01:01:51	1.04%	A	A	G
F*01:01:02:09	4.80%	C	T	T
F*01:01:02:10	20.53%	C	T	T
F*01:01:06	6.97%	A	A	T
F*01:05:01:02	1.88%	A	A	G

Previous studies have reported that the rs2523405‐T allele is significantly associated with HLA‐F protein expression in PBMCs from healthy donors, as well as with transcriptional expression levels [35]. To characterise the phasing of rs2523405 with HLA‐F alleles in the Japanese population, we analysed alleles with frequencies greater than 1.0%, using primers designed to amplify the genomic region encompassing rs2523405 (Figure 1B).

### 3.3 HLA‐G Diversity and UTR‐Defined Haplotypes

#### 3.3.1 HLA‐G Allele Distribution

Seven protein‐level *HLA‐G* alleles were observed in the Japanese population. The most frequent allele was G*01:01 (53.1%), followed by G*01:04 (45.4%) and 48.8% of individuals were heterozygous for G*01:01 and G*01:04.

At the four‐field resolution, the major constituent alleles within G*01:01 were G*01:01:01:01 (26.8%), G*01:01:02:01 (18.8%) and G*01:01:03:03 (5.1%), whereas G*01:04 was predominantly represented by G*01:04:01:01 (33.8%) and G*01:04:01:02 (11.1%), as shown in Table 4.

**TABLE 4:** HLA‐G allelic frequencies.

Allele	Number of observations (2n = 1062)	Frequency (2n = 1062)	GenBank accession number	IPD‐IMGT/HLA Database accession number	Status
G*01:01:01:01	285	26.84%	—	—	—
G*01:01:01:02	1	0.09%	—	—	—
G*01:01:01:05	5	0.47%	—	—	—
G*01:01:01:17	2	0.19%	OP765587	HWS10063374	Novel
G*01:01:01:18	1	0.09%	OP765590	HWS10063382	Novel
G*01:01:02:01	200	18.83%	—	—	—
G*01:01:02:06	1	0.09%	OP765591	HWS10063384	Novel
G*01:01:03:03	54	5.08%	—	—	—
G*01:01:30:01	15	1.41%	—	—	—
G*01:03:01:02	3	0.28%	—	—	—
G*01:04:01:01	359	33.80%	—	—	—
G*01:04:01:02	118	11.11%	OP902173	HWS10064491	Extended
G*01:04:01:06	1	0.09%	OP765592	HWS10063380	Novel
G*01:04:01:07	2	0.19%	OP765593	HWS10063386	Novel
G*01:04:01:10	2	0.19%	PP503419	HWS10070025	Novel
G*01:05:01 N	2	0.19%	—	—	—
G*01:06:01:01	5	0.47%	—	—	—
G*01:21 N	5	0.47%	OP765588	HWS10063376	Extended
G*01:43	1	0.09%	OP765589	HWS10063378	Novel

*Note:* ‐: Not‐Applicable.

Among common genotypes (frequency ≥ 1.0%), the most frequent were G*01:01:01:01/G*01:04:01:01 (17.3%), G*01:01:02:01/G*01:04:01:01 (13.6%) and G*01:01:01:01/G*01:01:02:01 (11.5%) (Table S3). Overall, 54.2% of individuals carried at least one copy of G*01:04:01:01.

A similar distribution was reported in a Japanese cohort of patients with acute graft‐versus‐host disease undergoing unrelated bone marrow transplantation, where G*01:01:01:01 (20.3%), G*01:01:02:01 (12.4%) and G*01:01:03:03 (4.7%) were predominant [28]. Likewise, analysis of the 1000 Genomes Project Japanese dataset (2n = 178) showed a comparable pattern, with G*01:01:01:01 (23.6%), G*01:01:02:01 (17.4%) and G*01:01:03:03 (3.4%) as the major four‐field alleles [34].

#### 3.3.2 Linkage of Four‐Field Alleles With UTR Haplotypes

Complete co‐segregation between the eight UTR‐defining SNPs and four‐field resolution HLA‐G alleles with ≥ 1% frequency was observed in the Japanese population, consistent with previous reports [36, 37]. The rs371194629 14‐bp deletion co‐segregated completely with G*01:04 alleles in this cohort.

In contrast, rs17875394 (g.–444 G > A), which is not included among these UTR‐defining 8 SNPs, exhibited the following pattern: the rs17875394‐G allele co‐segregated with G*01:01:01:01, G*01:01:02:01 and G*01:01:03:03, whereas for G*01:04:01:01 and G*01:04:01:02, both the rs17875394‐G and rs17875394‐A alleles were phased on these haplotype backgrounds, indicating no fixed co‐segregation. This pattern for G*01:04:01:01 and G*01:04:01:02 is consistent with Nishizawa et al. [27].

#### 3.3.3 3′ UTR 14‐Bp Indel–Defined Haplotypes

The newly designed HLA‐G primer set amplified both transcript regions (NM_001384290.1 and NM_001363567.2) and covered the 3′ UTR 14‐bp insertion/deletion (rs371194629) allowing four‐field allele and the exon 8 indel to be phased to specific four‐field alleles within single amplicons (Figure 1C). The rs371194629 14‐bp deletion co‐segregated completely with G*01:04 alleles in this cohort. In contrast, within G*01:01, G*01:01:01 was linked to the deletion, whereas G*01:01:02 and G*01:01:03 were linked to the insertion, indicating distinct 3′ UTR polymorphic structures within the same protein‐level allele group.

### 3.4 The HLA‐E, ‐F and ‐G Haplotype Analysis

**FIGURE 2:**
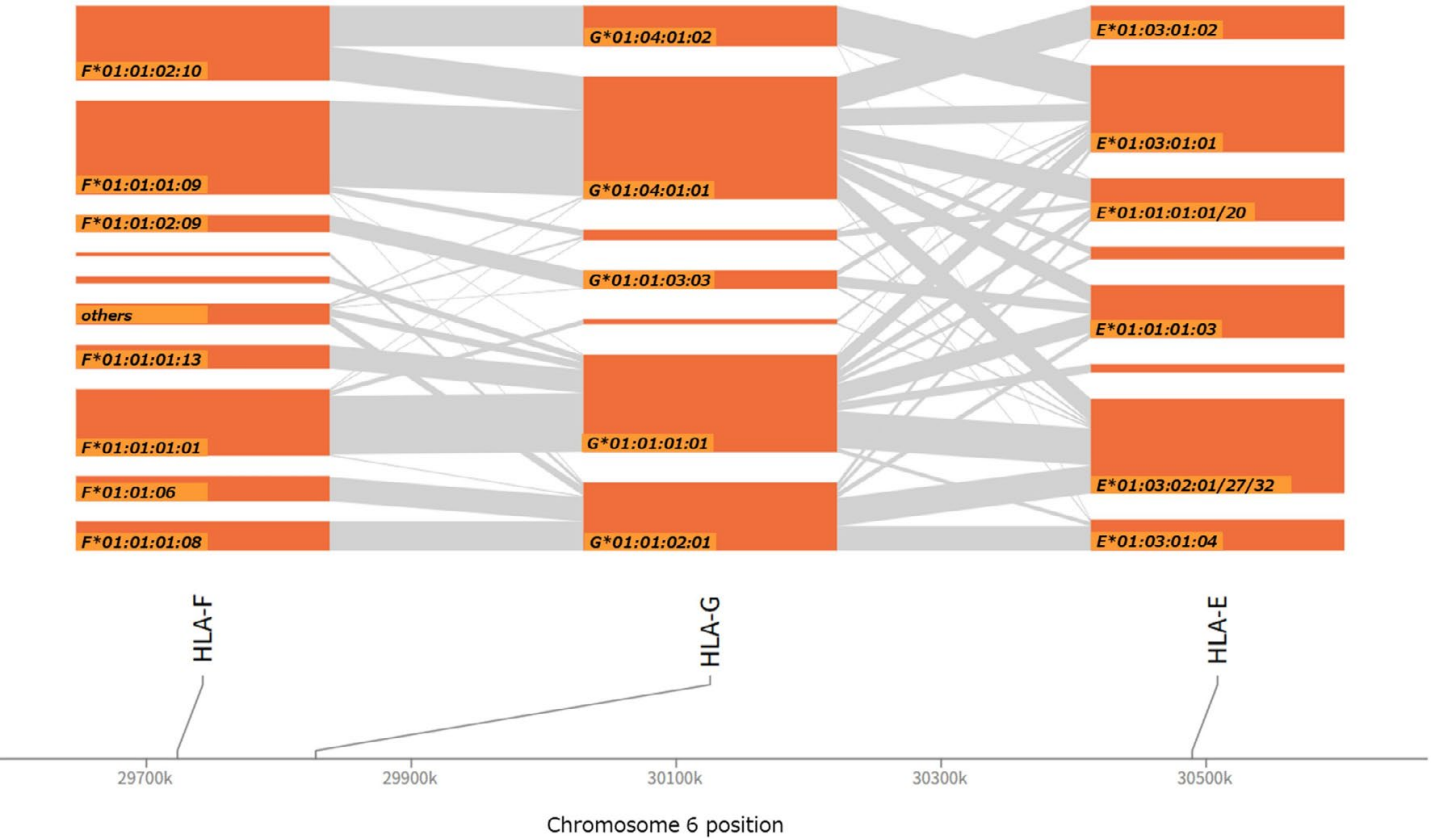
The positions of the bars correspond to the genomic locations of HLA‐E, HLA‐F and HLA‐G in the hg38 reference genome. Tiles within each bar represent four‐field alleles and segments connecting two alleles between adjacent genes represent haplotypes. The height of each tile and the width of each segment correspond to the frequencies of the respective HLA alleles and haplotypes.

No LD was observed between HLA‐E and either HLA‐F or HLA‐G, consistent with previous reports [18, 19], as shown in Figure 2. In the Japanese population, the three most frequent haplotypes across HLA‐E, HLA‐F and HLA‐G loci were F*01:01:02:10 ~ G*01:04:01:02 ~ E*01:03:01:01 (10.5%), F*01:01:02:10 ~ G*01:04:01:01 ~ E*01:03:01:02 (8.7%) and F*01:01:01:01 ~ G*01:01:01:01 ~ E*01:03:02:01 (8.2%), with closely concordant frequency estimates obtained using PyPop and BIGDAWG (Table S4).

**FIGURE 3:**
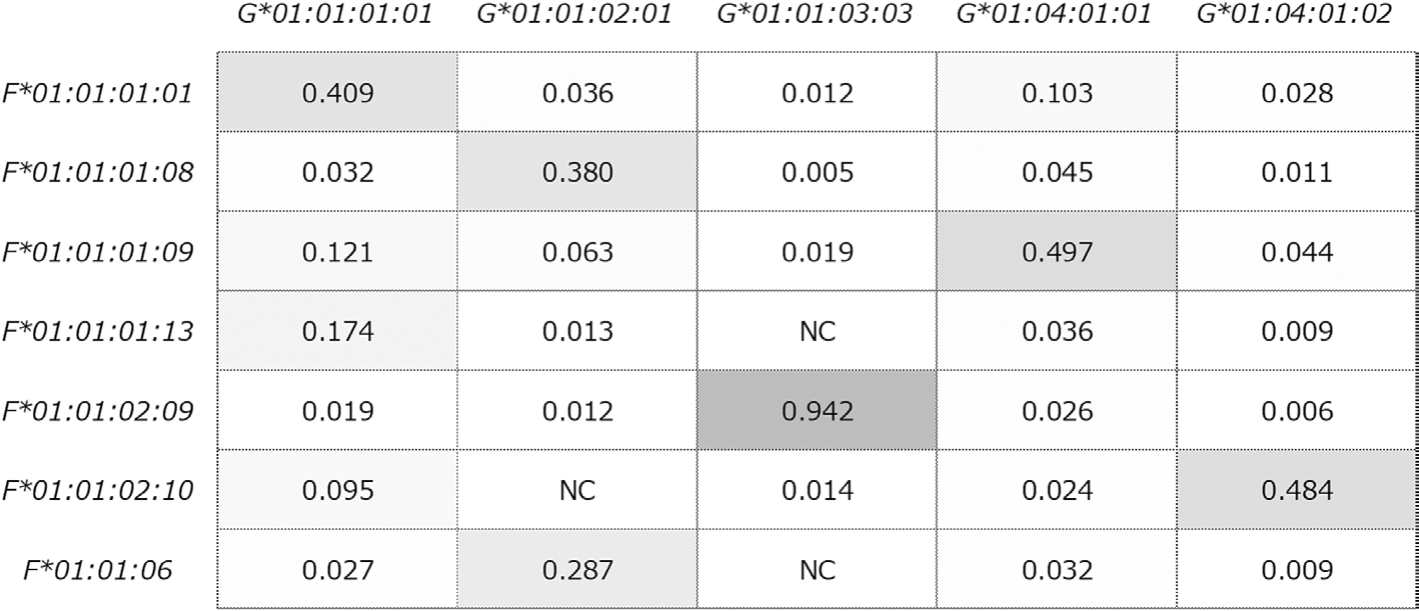
Heatmap of *r* ^2^ values between HLA‐F and HLA‐G alleles. The heatmap visualises linkage disequilibrium (LD) between HLA‐F and HLA‐G alleles observed at frequencies higher than 4%, using the *r* ^2^ metric. NC indicates values that were not calculated due to low allele frequency. Darker colours in the heatmap correspond to higher *r* ^2^ values.

Pairwise *r* ^2^ values between common HLA‐F and HLA‐G alleles are shown in Figure 3. The strongest LD signal was detected for the rare haplotype F*01:01:02:09 ~ G*01:01:03:03, which showed *D′* = 1.000 and *r* ^2^ = 0.942. In addition, moderate LD signals were observed for several haplotypes, including F*01:01:01:01 ~ G*01:01:01:01 (*D′* = 0.817, *r* ^2^ = 0.409), F*01:01:01:09 ~ G*01:04:01:01 (*D′* = 0.852, *r* ^2^ = 0.497) and F*01:01:02:10 ~ G*01:04:01:02 (*D′* = 1.000, *r* ^2^ = 0.484).

## 4 Discussion

In this work, we examined comprehensive full‐gene analyses of the non‐classical HLA Class I genes (HLA‐E, HLA‐F and HLA‐G) in 531 Japanese individuals. By using our newly designed primer sets, we are able to perform comprehensive HLA allele characterisation across the entire HLA‐Ib gene regions, including variants outside the IPD‐IMGT/HLA Database ‐registered core; we delineated the four‐field allele frequencies of the HLA‐Ib genes.

For HLA‐E, our results were consistent with previous observations showing that E*01:03 is the predominant allele in East Asians, including the large DKMS donor dataset [4]. At the protein‐level, E*01:01 and E*01:03 together accounted for 97.6% of the allele frequency in the Japanese population, and only three protein‐encoding alleles (E*01:01, E*01:03 and E*01:12) were detected.

For HLA‐F, we identified multiple noncoding four‐field alleles, even though protein‐altering polymorphisms were rare. This pattern suggests that regulatory variation, rather than amino acid sequence differences, may play a more prominent role in shaping HLA‐F biology. HLA‐F has been reported as an eQTL in neutrophils [38], and several SNVs have been associated with altered expression levels [35].

For HLA‐G, the strong haplotypic association between the 3′ UTR 14‐bp insertion/deletion polymorphism and certain HLA‐G alleles observed in this cohort underscores the tight linkage between coding and regulatory variation at this locus in the Japanese population.

The high‐resolution allele and haplotype data generated in this study provide a valuable reference for immunogenetic analyses in the Japanese population. For clinical transplantation, particularly haematopoietic cell transplantation, four‐field resolution characterisation of HLA‐Ib genes may improve donor selection by identifying population‐specific suballeles and regulatory haplotypes that are not captured when only protein‐level typing is performed.

At the protein‐level resolution, HLA‐E diversity in our cohort was limited. Notably, E*01:12 shares Arg at position 107 with E*01:01 but differs at position 276 in the α3 domain, where Pro is replaced by Gln, highlighting that rare protein variants can occur despite overall constrained protein‐level diversity. Within E*01:03, E*01:03:01 (42.0%) exceeded E*01:03:02 (26.9%), a pattern consistent with the geographic variation described by Lucas et al. [39]. Because E*01:03:01 is relatively uncommon outside Asia, higher‐resolution allele typing can reveal population‐specific patterns that are not apparent when classification is limited to the protein‐level distinction between E*01:01 and E*01:03. Although E*01:03:01 and E*01:03:02 are distinguished by rs1059510 (g.424C > T; codon 77), an SNV implicated in disease associations [40, 41], definitive functional evidence remains lacking.

At four‐field resolution, the overall allele distribution in our cohort differs from that reported in other populations. For example, E*01:03:02:01/27/32 accounted for 26.1% and was the most frequent allele in this study, whereas E*01:01:01:01/20 accounted for 11.8% and was the fourth most frequent allele (Table 1). In contrast, in an Anthony Nolan registry cohort comprising transplant patients and unrelated donors, E*01:01:01:01 was the most frequent allele (56.9%), followed by E*01:03:02:01 (29.9%) [6]; similarly, in the Beninese Toffin population, E*01:01:01:01 (40.0%) and E*01:03:02:01 (24.7%) were the two most frequent alleles [17].

At the locus scale, promoter, 3′ UTR and downstream SNVs did not co‐segregate with four‐field *HLA‐E* alleles in our cohort, consistent with Ramalho et al. [34] and with the report by Pyo et al. [19] that LD between intragenic *HLA‐E* variants and flanking SNVs may be limited. Together with the absence of novel exonic variants, these findings reinforce the notion that *HLA‐E* exhibits limited protein‐level diversity and support a model in which regulatory variants and high‐resolution coding haplotypes can evolve at least partly independently in the Japanese population.

In this study, the frequency of F*01:01 was comparable to that previously reported in East Asian populations, whereas F*01:03 (which differs from F*01:01 by p.S251P in the α3 domain) has been reported to exceed 10.0% in several non‐Asian populations, yet remained below 1.0% in this Japanese cohort, consistent with previous observations [7, 35]. At the three‐field resolution, the major alleles were F*01:01:01 (64.2%), F*01:01:02 (25.5%) and F*01:01:06 (7.0%). The rs2076183‐A variant defining F*01:01:02 has been reported at 29.2% in the 1000 Genomes East Asian population [7] and 19.7% in the jMorp/ToMMo 61KJPN dataset [41], in line with the relatively high frequency of F*01:01:02 in our cohort.

At four‐field resolution, the Japanese distribution shared some features with that reported in other cohorts. In a study predominantly of European ancestry, the most frequent alleles were F*01:01:01:01 (21.2%), F*01:03:01:01 (20.7%) and F*01:01:01:09 (20.2%) [18], whereas in a Brazilian cohort, F*01:01:01:09 (17.4%), F*01:01:01:08 (17.1%) and F*01:01:01:01 (14.5%) were among the most common, with F*01:03:01:01 also frequent (10.2%) [16]. Across these studies and our Japanese cohort, F*01:01:01:01, F*01:01:01:08 and F*01:01:01:09 were recurrently observed among the more frequent alleles, whereas F*01:03:01:01 was not among the most frequent in Japanese individuals.

Despite limited protein altering polymorphism, we identified multiple noncoding four‐field alleles, supporting the possibility that regulatory variation, rather than amino acid differences, contributes substantially to HLA‐F biology. Consistent with this, HLA‐F has been reported as an eQTL in neutrophils [38], and several SNVs have been associated with altered expression [35]. The presence of multiple transcript isoforms (e.g., transcripts lacking exon 4 or incorporating an alternative exon 7) further raises the possibility of isoform‐specific functions. Our primer design enabled sequencing across exon 7 of NM_001098479.2, revealing variants encoding a p.353 substitution in alleles such as F*01:01:02:09 and F*01:01:02:10 (Table 3). Previous evidence of HLA‐F isoform expression in breast cancer tissue supports the biological relevance of transcript diversity [42]. Together, these findings highlight the importance of integrating allele‐level, regulatory‐region and isoform‐level information to understand HLA‐F function.

Population differences at classical HLA loci may also contribute to cross‐population differences at HLA‐F through LD. For example, prior work reported LD between F*01:03:01 and A*03:01:01 (*r* ^2^ = 0.679) and between F*01:01:02 and A*11:01:01 (*r* ^2^ = 0.401) [18]. In the jMorp/ToMMo 61KJPN dataset, A*03:01 is rare (0.5%), whereas A*11:01 is more common (8.3%) [43], consistent with our observation that F*01:03:01 is rare while F*01:01:02 is relatively frequent in Japanese individuals.

For HLA‐G, G*01:01 (53.1%) and G*01:04 (45.4%) predominated, broadly concordant with the jMorp/ToMMo 61KJPN frequencies (56.9% and 42.0%, respectively) [43]. The high prevalence of G*01:04 among Japanese individuals aligns with its elevated frequency in East Asians (e.g., 25.1% in the 1000 Genomes East Asian population [7]), although within‐Japan variation has been reported (e.g., 39.7% vs. 58.9% in an aGVHD cohort [28]). The higher G*01:04 proportion reported by Suzuki et al. can likely be attributed to their calculation based on 338 pairs (676 individuals) [28], whereas our estimates reflect allele frequencies.

Regarding splice‐site–proximal variation, Ribeyre et al. predicted that g.1827G > A (at the end of exon 4) creates an exonic cryptic acceptor site [36], but we did not detect g.1827A in this cohort, suggesting that this variant is rare in the Japanese population. Similarly, although g.99G > A (intron 2) and g.494A > C (intron 3) can disrupt intronic cryptic acceptor sites, only G*01:01:01:05, which preserves no disrupted sites at both positions, was identified, consistent with reports that this allele is less common in Japanese compared with Caucasians. Overall, cryptic acceptor–disrupting variants in *HLA‐G* differ substantially by ethnicity, which likely explains the absence of certain variants in the Japanese population.

For HLA‐G, population‐level comparisons at four‐field resolution have already been reported in previous studies [34], and our results were largely consistent with those observations. Moreover, differences in the distribution of cryptic acceptor–disrupting variants across ethnic groups suggest population‐specific HLA‐G regulatory mechanisms. Because these variants can influence splicing efficiency or transcript stability, their absence or presence may contribute to lineage‐ or tissue‐specific patterns of HLA‐G expression. The strong haplotypic association between the 3′ UTR 14‐bp insertion/deletion polymorphism and certain HLA‐G alleles observed in our cohort further underscores the tight linkage between coding and regulatory variation at this locus in the Japanese population. Collectively, these patterns suggest that HLA‐G diversity may be shaped less by amino acid–altering changes and more by variation affecting mRNA processing and expression.

The high‐resolution allele and haplotype data generated in this study provide a valuable reference for immunogenetic analyses in the Japanese population. For clinical transplantation, particularly haematopoietic cell transplantation, four‐field resolution characterisation of HLA‐Ib genes may improve donor selection by identifying population‐specific suballeles and regulatory haplotypes that are not captured when only protein‐level typing is performed. Differences in the frequencies of alleles such as E*01:03:01, F*01:01:02 and G*01:04 highlight the importance of population‐tailored reference datasets when evaluating risks associated with HLA‐Ib–mediated immune modulation, as described in the recent comprehensive review by Lucas et al. [39], which details how population‐specific HLA‐E variation can influence haematopoietic cell transplantation outcomes.

From a research‐infrastructure perspective, the four‐field allele reference presented here expands population‐specific resources for Japanese cohorts that have been underrepresented in global datasets. The comprehensive high‐resolution HLA‐Ib allele data generated for the Japanese population in this study will support precision medicine efforts and facilitate future epidemiological, evolutionary and functional research in East Asian populations.

As demonstrated by the Japanese frequency data generated in this study, HLA‐Ib genes exhibit greater polymorphism at the four‐field level than previously appreciated. The dataset presented here will provide a valuable reference for future disease association studies in the Japanese population and will also contribute to evolutionary research and broader immunogenetic investigations. Moreover, extending these analyses to include LD structures with classical HLA‐Ia genes will be important for a more comprehensive understanding of HLA haplotype architecture.

## Author Contributions

Ikue Ito‐Naito designed the study. Katsushi Tokunaga, Seik‐Soon Khor and Yosuke Omae supervised the study. Ikue Ito‐Naito, Tetsuya Sato and Shunichi Wakabayashi developed and tested the primer sets. Daisuke Kinoshita and Naoya Kubo contributed to data acquisition, analysis and interpretation. Ikue Ito‐Naito and Seik‐Soon Khor identified and submitted the novel alleles. Ikue Ito‐Naito wrote the first draft of the manuscript and served as the corresponding author. Hiroshi Hayashi supervised the research and contributed to the critical revision of the manuscript. All authors contributed to manuscript revision and approved the final version.

## Ethics Statement

The study protocol was reviewed and approved by the Ethics Committee of the Japanese Institute for Human Sciences (JIHS) and by the Ethics Committee of H.U. Group Holdings Inc.

## Conflicts of Interest

Ikue Ito‐Naito, Tetsuya Sato, Shunichi Wakabayashi, Naoya Kubo, Daisuke Kinoshita, and Hiroshi Hayashi are employees of H.U. Group Research Institute G.K. Ikue Ito‐Naito, Seik‐Soon Khor, Tetsuya Sato, Shunichi Wakabayashi, Yosuke Omae, and Katsushi Tokunaga are named as inventors on patents related to the methods described in this study, including Japanese Patent No. 7863825 and the corresponding PCT application (PCT/JP2021/037121; WO2022075403A1).

## Supporting information


**Figure S1:** Data analysis workflow. A schematic overview of the analytical pipeline used for HLA allele assignment, validation, and identification of novel or extended alleles.

**Table S1:** Genotype distribution of HLA‐E in the Japanese population.
**Table S2:** Genotype distribution of HLA‐F in the Japanese population.
**Table S3:** Genotype distribution of HLA‐G in the Japanese population.
**Table S4:** HLA‐F~G~E haplotype frequencies (≥ 2%) in the Japanese population.

## Data Availability

The datasets generated and analysed during the current study are available from the corresponding author on reasonable request.
