# Supplementary material for: Genetic Diversity and Haplotype Architecture of Non‐Classical HLA Class I Genes in the Japanese Population
Source: HLA. 2026 Aug 10;108(2):e70898. doi: 10.1111/tan.70898 (PMC13457674; doi:10.1111/tan.70898)
Supplement: Supplementary file 1 [file TAN-108-e70898-s001.docx]

**Supplementary Methods**

1.1. Design of *HLA-E, HLA-F, and HLA-G* primers for long-range PCR

One set of primers was designed for each of the genes by executing PCR primer design tool Primer 3 on regions stored in a human genome reference sequence (genome assembly version hg 38) and Alternate sequences.

To assess whether the designed primer set could comprehensively PCR-amplify HLA alleles, we performed an *in silico* analysis using whole-genome sequencing (WGS) data. WGS data from the following two populations: Tokyo Healthy Control (THC) and the HipSci resource were downloaded from the European Nucleotide Archive (ENA; study accession PRJEB15299, <https://www.ebi.ac.uk/ena>) and　used [1]. THC samples were taken from healthy Japanese individuals residing in the Tokyo area. Whole-genome sequencing was performed using Illumina sequencing platforms. The HipSci samples consisted of individuals of European descent [2].

For each sample, reads derived from HLA genes and their flanking regions were extracted using BWA v0.7.17 for alignment and samtools v1.10 for filtering. HLA allele calling was performed using HLA-HD v1.2.1 on the filtered FASTQ files.

Consensus sequences for HLA-Ib genes and adjacent regions were generated using freebayes v1.3.1, WhatsHap v1.0, and bcftools v1.10.2, and correlated with the HLA allele information of each sample. Primer binding sites were evaluated on these consensus sequences using Bowtie2 v2.3.5.1.

No mismatches were observed in either the forward or reverse primers of the designed primer sets across all THC alleles (2n = 742) and HipSci alleles (2n = 290), confirming that the designed primers achieve high allele comprehensiveness.

1.2. First PCR conditions for *HLA-E, HLA-F*, and *HLA-G* amplification

The *HLA-E* locus was amplified as a single amplicon of approximately 8.4 kb (Figure 1A), spanning from the extended promoter region to the complete 3’ UTR, using primers modified at the 5’ end with an amino modifier C6 (/5AmMC6/). PacBio universal sequencing (in lowercase) were added to the 5’ end of each primer. The forward primer was /5AmMC6/gcagtcgaacatgtagctgactcaggtcacCCGGCCTGGAGAAATTCAGT, and the reverse primer was /5AmMC6/tggatcacttgtgcaagcatcacatcgtagCCCAAGAAAACAGTGCCACG. Each 25 μl PCR mixture contained KOD One PCR Master Mix (TOYOBO CO., LTD., Osaka, Japan), 0.30 μM of each primer, and 5 ng of genomic DNA. PCR cycling conditions were as follows: initial denaturation at 94 °C for 1 min; 5 cycles of 98 °C for 10 s and 74 °C for 3 min; 5 cycles of 98 °C for 10 s and 72 °C for 3 min; 5 cycles of 98 °C for 10 s and 70 °C for 3 min; followed by 25 cycles of 98 °C for 10 s and 68 °C for 3 min; with a final hold at 4 °C.

The *HLA-F* locus (approximately 4.8 kb; Figure 1B) and the *HLA-G* locus (approximately 4.9 kb; Figure 1C) were amplified as single amplicons spanning from the 5’ UTR to 3′ UTR, including untranslated regions harboring variants associated with expression levels. For both loci, primers were modified at the 5’ end with an amino modifier C6 (/5AmMC6/), and PacBio universal sequencing tags (in lowercase) were added to the 5’ end of each primer, as described for H*LA-E*. The primer sequences were as follows: for *HLA-F*, the forward primer was /5AmMC6/gcagtcgaacatgtagctgactcaggtcac CAAAAGAGTCCGGGGAGTCC and the reverse primer was /5AmMC6/tggatcacttgtgcaagcatcacatcgtag AAAGCTCGTGAGGAATGGGG; for *HLA-G*, the forward primer was /5AmMC6/gcagtcgaacatgtagctgactcaggtcac GCACTAGTGAGGGGCATTGT and the reverse primer was /5AmMC6/tggatcacttgtgcaagcatcacatcgta GACCTCTCCTCTCGGGAAGT. For *HLA‑F*, the primer set was designed to amplify the full‑length locus encompassing the UTRs corresponding to transcript variants NM_018950.3 and NM_001098479.2 (Figure 1B). A primer set specific for *HLA‑G* was designed to amplify both transcript regions corresponding to NM_001384290.1 and NM_001363567.2 (Figure 1C). The primer set was further configured to include the 14‑bp deletion/insertion polymorphism in exon 8 (rs371194629), a variant known to influence alternative splicing and transcript stability [3, 4]. In addition, the primers were constructed to enable low‑resolution UTR typing (UTR 1–8) by targeting the eight SNPs described by Ribeyre et al [5]. and Di Cristofaro et al. (−725/rs1233334, −716/rs2249863, −201/rs1233333, −56/rs17875397, exon 8 ins/del/rs66554220, 3142/rs1063320, 3187/rs9380142, and 3196/rs1610696) [6].

A separate PCR reaction was performed for each primer set using PrimeSTAR GXL Premix (2X) (Takara Bio Inc., Shiga, Japan), 0.20 μM of each primer, and 5 ng of genomic DNA. Cycling conditions were: initial denaturation at 94 °C for 1 min, followed by 30 cycles of 98 °C for 10 s and 68 °C for 5 min, with a final hold at 4 °C.

1.3. Library preparation, and sequencing of *HLA-E, HLA-F, and HLA-G*

First PCR products were purified using Sera-Mag Select (Cytiva, Marlborough, MA, USA) and eluted with 25 μL of Buffer EB (QIAGEN, Hilden, Germany). A second PCR with barcoding was performed using PacBio Barcoded Universal Primers (BUP) and was using the single molecule real-time (SMRT) sequencing technology (Pacific Biosciences of California, Inc., Menlo Park, CA, USA). For the second PCR of *HLA-E*, each 25 μL reaction contained KOD One PCR Master Mix (TOYOBO CO., LTD., Osaka, Japan), 0.30 μM of mixed BUP, and 5 μL of the first PCR product. Cycling conditions were: initial denaturation at 94 °C for 1 min, followed by 10 cycles of 98 °C for 10 s and 68 °C for 3 min, with a final hold at 4 °C. For the second PCR of *HLA-F* and *HLA-G*, each 25 μL reaction contained PrimeSTAR GXL Premix (2X) (Takara Bio Inc., Shiga, Japan), 0.20 μM of mixed BUP, and 5 μL of the first PCR product. Cycling conditions were: initial denaturation at 94 °C for 1 min, followed by 10 cycles of 98 °C for 10 s and 68 °C for 5 min, with a final hold at 4 °C.

Libraries for sequencing were constructed from pooled DNA for each sample using the SMRTbell Express Template Prep Kit (Pacific Biosciences of California, Inc., Menlo Park, CA, USA) according to the manufacturer’s instructions. Sequencing was performed on the PacBio Sequel or Sequel IIe system (Pacific Biosciences of California, Inc., Menlo Park, CA, USA). Subreads were generated from raw polymerase reads, and circular consensus sequencing (CCS) reads were produced using SMRT Link software with the heteroduplex algorithm (hd-finder) option enabled. Candidates for novel or extended alleles were validated by preparing libraries from the initial PCR products and sequencing them on a MiSeq system (Illumina, San Diego, CA, USA) using 251 bp paired-end reads with the MiSeq Reagent Kit v2, following the manufacturer’s instructions.

Supplementary References

1. D'Antonio M, Reyna J, Jakubosky D, et al. Systematic genetic analysis of the MHC region reveals mechanistic underpinnings of HLA type associations with disease. Elife. 2019;8:e48476. Published 2019 Nov 20. doi:10.7554/eLife.48476
2. Kilpinen H, Goncalves A, Leha A, et al. Common genetic variation drives molecular heterogeneity in human iPSCs. Nature. 2017;546(7658):370-375. doi:10.1038/nature22403
3. Rousseau P, Le Discorde M, Mouillot G, Marcou C, Carosella ED, Moreau P. The 14 bp deletion-insertion polymorphism in the 3' UT region of the HLA-G gene influences HLA-G mRNA stability. Hum Immunol. 2003;64(11):1005-1010. doi:10.1016/j.humimm.2003.08.347
4. Hviid TV, Rizzo R, Melchiorri L, Stignani M, Baricordi OR. Polymorphism in the 5' upstream regulatory and 3' untranslated regions of the HLA-G gene in relation to soluble HLA-G and IL-10 expression. Hum Immunol. 2006;67(1-2):53-62. doi:10.1016/j.humimm.2005.12.003
5. Ribeyre C, Carlini F, René C, et al. HLA-G Haplotypes Are Differentially Associated with Asthmatic Features. Front Immunol. 2018;9:278. Published 2018 Feb 23. doi:10.3389/fimmu.2018.00278
6. Di Cristofaro J, El Moujally D, Agnel A, et al. HLA-G haplotype structure shows good conservation between different populations and good correlation with high, normal and low soluble HLA-G expression. Hum Immunol. 2013;74(2):203-206. doi:10.1016/j.humimm.2012.10.027

Supplementary Figure S1

Data analysis workflow. A schematic overview of the analytical pipeline used for HLA allele assignment, validation, and identification of novel or extended alleles.
